# Lateralized Reverse Shoulder Arthroplasty vs. Medialized Design with Latissimus Dorsi Transfer for Cuff Tear Arthropathy with Loss of External Rotation and ER Lag Sign

**DOI:** 10.3390/jcm14165679

**Published:** 2025-08-11

**Authors:** Mara Warnhoff, Philipp Moroder, Laurent Audigé, Giovanni Spagna, Yacine Ameziane, Tim Schneller, Markus Scheibel, Florian Freislederer

**Affiliations:** 1 Schulthess Clinic, 8008 Zurich, Switzerland; 2 Charité Universitaetsmedizin Berlin, 10117 Berlin, Germany

**Keywords:** cuff tear arthropathy, reverse shoulder arthroplasty, latissimus dorsi transfer, lateralized prosthesis, ER lag sign, shoulder function outcomes

## Abstract

**Background**: The management of irreparable posterosuperior rotator cuff tears with an isolated loss of external rotation presents significant challenges. Latissimus dorsi tendon transfer in conjunction with medialized reverse total shoulder arthroplasty has been employed to rectify external rotation deficits; however, lateralized RTSA designs may yield similar outcomes with a reduced incidence of complications. The objective of this study was to compare the clinical outcomes of lateralized reverse total shoulder arthroplasty without latissimus dorsi tendon transfer against medialized RTSA with LDT in patients with ILER and a positive external rotation lag sign. **Methods**: This retrospective cohort study involved 34 patients diagnosed with CTA and severe external rotation deficiency, characterized by a positive ER lag sign and 0° active ER. The patients were treated with either lateralized reverse total shoulder arthroplasty (*n* = 21) or medialized RTSA with LDT (*n* = 13). Outcomes evaluated preoperatively and at the 24-month follow-up comprised range of motion, ER lag sign, Constant–Murley Score, SPADI, and radiographic offset parameters. Statistical analyses were adjusted for age, sex, and baseline values. **Results**: At follow-up, 70% of patients undergoing lateralized RTSA exhibited resolution of ER lag, compared to 23% in the LDT group (*p* < 0.05). Active external rotation improvement was more significant in the LDT group (34.6° compared to 18.5°, *p* < 0.05). However, both groups exhibited comparable final external rotation and functional scores (CMS: 63 ± 9 vs. 63 ± 16; SPADI: 73 ± 20 vs. 74 ± 22). Lateralized RTSA demonstrated superior preservation of internal rotation, as evidenced by a higher percentage of patients achieving a negative Apley scratch test (67% compared to 23%, *p* < 0.05). A greater glenoidal offset correlated with improved postoperative external rotation and resolution of external rotation lag. The influence of teres minor integrity was more significant in the LDT group. **Conclusions**: Lateralized reverse total shoulder arthroplasty without latissimus dorsi tendon transfer provides similar functional restoration of external rotation in irreparable posterosuperior rotator cuff tear patients, accompanied by reduced complications, shorter surgical durations, and improved preservation of internal rotation. LDT has the potential to provide enhanced ER gains from a low baseline; however, it is characterized by increased invasiveness and technical complexity. Prosthetic lateralization is a biomechanically effective method for restoring external rotation in patients with rotator cuff arthropathy and external rotation deficits.

## 1. Introduction

For active patients without severe degenerative changes, anatomic rotator cuff repair is the preferred treatment for posterosuperior rotator cuff tears involving the supraspinatus and infraspinatus tendon [1]. The optimal management of irreparable posterosuperior rotator cuff tears (IPRCTs) remains a topic of debate among shoulder surgeons. Although patients can maintain good function with nonoperative treatment, loss of external rotation (ER) detrimentally impacts their functional capacity, as this movement plays a crucial role in activities of daily living [2]. The optimal treatment for IPRCTs remains controversial and there are various options, including conservative approaches, arthroscopic interventions, tendon transfers, and reverse total shoulder arthroplasty (RTSA).

While RTSA using the Grammont Design has been effective for addressing elevation deficits due to irreparable rotator cuff tears, it has been less efficacious in restoring active ER [3,4]. Latissimus dorsi tendon transfer (LDT) had already been introduced for the treatment of IPRCTs by Gerber et al. in 1988 [5]. It later solidified its efficacy in reinstating active ER in cases of IPRCT, isolated or with teres major transfer (TMT), as a modified l’Episcopo technique, as well as combined with RTSA, as reported by Gerber et al. and Boileau et al. [6,7]. Advancements in prosthetic design, featuring a lateralized center of rotation, have shown the potential to enhance overall range of motion, also leading to improved active ER by retensioning the remaining rotator cuff and recruiting posterior deltoid fibers through better deltoid wrapping, increasing abduction strength, and decreasing the likelihood of scapular notching [8,9,10,11]. This approach may potentially minimize surgery time and complications compared to performing RTSA with LDT.

Prior studies have focused on individuals exhibiting significant external rotation deficits marked by MRI-verified muscle atrophy, inability to actively elevate, and total loss of external rotation accompanied by a positive horn blower sign, referred to as “CLEER”: combined loss of active elevation and ER [6]. However, the treatment options for improving external rotation in patients with isolated significant external rotation weakness who do not meet the strict CLEER criteria but qualify for a reverse total shoulder arthroplasty (RTSA) remain unclear. 

These patients typically exhibit an ER lag sign [12] preoperatively, known as “ILER” (isolated loss of ER [6]). The aim of this retrospective cohort study was to compare two interventions for “ILER” patients with a positive ER lag sign: lateralized RTSA and medialized RTSA with LDT.

## 2. Materials and Methods

Outcomes of patients undergoing RTSA between September 2007 and December 2018 were extracted from a local institutional database. The study only included patients who had rotator cuff tear arthropathy (CTA) and could barely move their arm outward before surgery (showing a positive lag sign and having a maximum active external rotation of 0°). Patients were divided into two groups: The first group (group L; *n* = 21) was treated with the Univers Revers Total Shoulder Arthroplasty (Arthrex Inc., Naples, FL, USA), a lateralized prosthesis with a 135° neck-shaft-angle (NSA). The second group (group T; *n* = 13) underwent surgery using the PROMOS Reverse Modular Shoulder System (Smith and Nephew, Memphis, TN, USA) combined with LDT. All surgeries were performed at the same specialized orthopedic hospital using a standardized surgical approach and postoperative rehabilitation protocol. The subscapularis muscle was repaired in 92.3% of cases in group T and 95.2% in group L.

Patients were evaluated both preoperatively and at follow-up (24 months postoperatively) for the following outcomes: anterior elevation, abduction, ER, the Apley scratch test, the ER lag sign, abduction strength, the Constant–Murley Score (CMS), and the Shoulder Pain and Disability Index (SPADI) (Table 1 and Table 2). All range-of-motion parameters were assessed both actively and passively.

The Apley scratch test, often referred to as Dawbarn’s test, provides a rapid assessment of a patient’s active range of motion, involving medial rotation, extension, and adduction. During the Apley scratch test, the patient attempts to reach their hand up their back, aiming to position it between the shoulder blades, starting below.

To detect an ER lag in our study, the patient was positioned standing, with the elbow flexed at a 90-degree angle. The examiner passively put the shoulder in near-maximal external rotation, at around 20° of abduction and maximal external rotation, while supporting both the elbow and the wrist. After positioning the arm, the examiner relinquished support of the wrist while still stabilizing the elbow and asked the patient to actively sustain the externally rotated posture. The test was marked as positive if the patient was unable to sustain the position and the arm deviated into internal rotation; this signified a positive external rotation lag sign. The degree of lag was recorded when applicable, although it was not quantified with a standardized scale.

The Constant–Murley Score (CMS) is a prevalent instrument for evaluating comprehensive shoulder functionality. It is a 100-point scale that assesses pain, activities of daily living (ADL), range of motion (ROM), and strength. The CMS is regarded as an essential instrument for monitoring alterations in shoulder functionality over time.

The Shoulder Pain and Disability Index (SPADI) is a patient-reported questionnaire designed to assess pain and functional impairments of the shoulder. The instrument comprises 13 items, categorized into two subscales: Pain (5 items) and Disability (8 items). Each item is evaluated using a visual analog scale (VAS) ranging from 0 to 10. The data are subsequently transformed into percentages, where 0 signifies optimal performance and 100 denotes the poorest performance.

Preoperative radiologic evaluations encompassed the assessment of fatty infiltration of the rotator cuff utilizing the Goutallier scale and the grading of CTA according to Hamada and Fukuda, based on MRI and AP-view X-rays [13,14] (Table 1). All patients received preoperative MRI imaging.

Lateralization was quantified through the measurement of radiographic prosthetic offset parameters, specifically glenoidal offset, glenosphere offset, and humeral offset, assessed on postoperative anteroposterior (AP) images (Table 1).

Data were stored utilizing the REDCap (Research Electronic Data Capture) system and subsequently exported for statistical analysis with Intercooled Stata version 17 (StataCorp LP, College Station, TX, USA). Comparative analyses using linear regression models adjusted for age, sex, and the preoperative value of the model’s dependent variable for the 2-year follow-up were conducted. Random-intercept linear mixed models, each controlling for age and the preoperative value of the model’s dependent variable, were established to take repeated measurements into account. The level of significance was set at 0.05.

The main objective of this study was to examine the incidence of the ER lag sign following the implantation of a medialized prosthesis with an LDT, in contrast to the implantation of a lateralized prosthesis without an LDT.

## 3. Results

This analysis included a total of 34 patients. Thirteen patients were treated with a medialized prosthesis and an LDT, while twenty-one patients were treated with a lateralized shoulder arthroplasty. The mean age at surgery was 74.3 years, and 65% were females. Table 1 provides the demographics and descriptive characteristics of the analyzed cohort.

Regarding the most important characteristics, the groups were comparable, i.e., did not show significant differences. Overall, six surgeons were involved in the treatment of these patients; however, the bulk of surgeries (88%, 30 cases) were performed by only two surgeons, each of whom treated patients from both groups.

**Figure 1:**
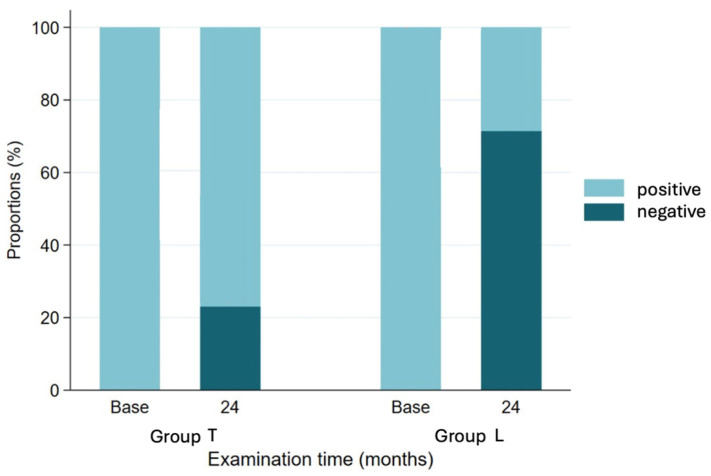
Presence of external rotation lag sign at baseline and 24-month follow-up of two groups.

At the 24-month follow-up, 70% of patients in group L (15 patients) no longer showed an ER lag, compared to 23% in group T (3 patients) (*p* < 0.05; see Figure 1).

**Figure 2:**
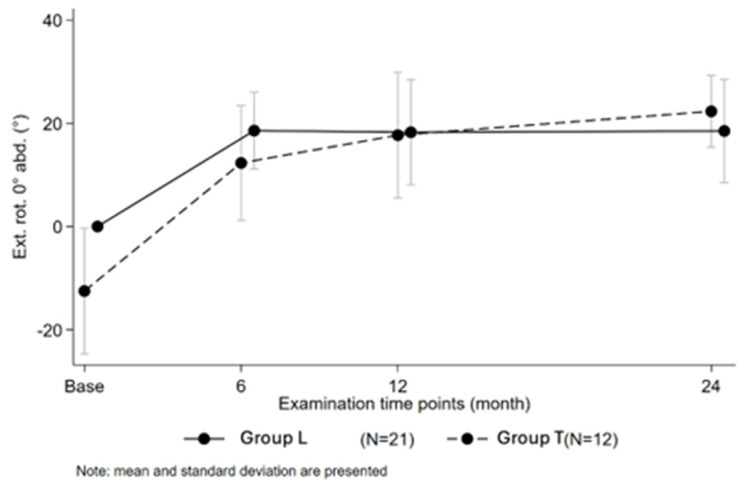
Development of external rotation at 0° abduction over time of two groups.

In contrast, mean active ER at follow-up in group L was 19° ± 10 (range: 0–30°) and 22° ± 7 (range: 10–30°) in group T (*p* = 0.35) (Figure 2). Change from baseline for active ER in group L was 18.5° ± 9.5 (range: 0–30°) and 34.6° ± 13.1 (range: 10–55°) in group T (*p* < 0.05 *). Average internal rotation in group L was 117° ± 26 (range: 90–160°) and 129° ± 26 (range: 70–160°) in group T (*p* = 0.159). A negative Apley scratch test (active internal rotation of Th3 or more) was apparent in 67% of patients in group L at 24-month follow-up and 23% of patients in group T (*p* < 0.05 **). Mean Constant–Murley Score in group L amounted to 63 points ± 9 (range: 45–73 points) and 63 points ± 16 (range: 29–83 points) in group T (*p* = 0.93). Average SPADI at follow-up was 74 points ± 22 (range: 23–99 points) for group T and 73 points ± 20 (range: 25–91 points) for group L (*p* = 0.9). Postoperative clinical outcomes at 24-month follow-up of the analyzed cohorts can be looked up in Table 2.

Prosthetic offset parameters (glenoidal offset, glenosphere offset, and humeral offset) were assessed using postoperative AP-view X-rays. Mean medialization of the center of rotation in group L was 20.4 mm ± 4.4 (range: 14.6–27.9 mm) and 23.7 mm ± 2.8 (range: 19.4–29.1 mm) in group T (*p* < 0.05 *). All offset parameters can be found in Table 1. Most offset parameters were significantly greater in patients exhibiting postoperative resolution of the ER lag sign, including glenoidal offset (*p* < 0.05) and humeral offset (*p* < 0.05) (Table 3). Additionally, we noted that a larger glenoidal offset was associated with better postoperative ER.

**Figure 3:**
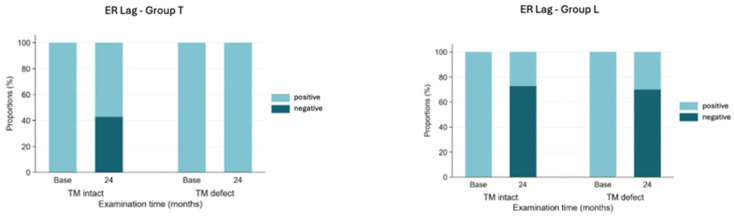
Presence of external rotation lag sign at baseline and 24-month follow-up for two groups according to status of teres minor.

Regarding active ER, patients with a defective teres minor muscle had a mean change of 26.4° regardless of prior surgery, and the group with an intact teres minor had a mean change of 18.7° pre-/postoperatively. The difference in delta pre-/post-ER (after 24 months) between the two states of the teres minor, regardless of prior surgery, was highly significant at 7.7° (unpaired *t*-test, *p* < 0.05). There was a highly significant difference in ER lag pre-/postoperatively (24 m) for group T regarding the state of the teres minor (McNemar’s chi-squared test, *p* < 0.05) (Figure 3).

Patients with an intact teres minor were more likely to have a negative ER lag sign 24 months postoperatively than those with a defective teres minor (*p* < 0.05, Fisher’s exact test). In patients with an intact teres minor muscle, a higher proportion of group T patients (40%) had a negative ER lag, while in group L, an intact teres minor had less impact on ER lag 24 months after surgery. If the teres minor muscle was defective, none of the group T patients had a negative ER lag postoperatively (Figure 3).

## 4. Discussion

The aim of this study was to compare lateralized RTSA and medialized RTSA with LDT for “ILER” patients with a positive ER lag sign. We found that lateralized RTSA is almost as effective in treating ER deficit as a medialized RTSA with tendon transfer, while it shows significantly less loss of internal rotation and saves time in the OR [15].

While the use of additional tendon transfer in combination with RTSA has been explored to address ILER, it is not without controversy. This arises from several factors. Firstly, the additional procedure increases the overall surgery time, which can be a concern, especially in older or medically complex patients [15]. Secondly, it poses a risk of infection, and the procedure itself is technically demanding, requiring a high level of surgical expertise. Moreover, there is a risk of potential nerve injury during the transfer, as well as the creation of stress risers in bone from drill holes [16]. Additionally, there is a concern about potential loss of internal rotation (IR) or decreased potential IR gains, which can affect patients’ overall shoulder function [17]. Revision rates for such procedures have been reported in up to 24% of cases [18], indicating the complexity and variability of outcomes. In 2018, Flury et al. conducted a study analyzing patients with an ER deficit who received RTSA, with or without latissimus dorsi transfer [15]. The findings of this study indicated that patients with LDT exhibited a significant internal rotation deficit, with 77% unable to reach the lumbosacral region compared to 46% of control patients (*p* < 0.05). Additionally, they had on average a 26 min (*p* < 0.05) longer operating time with a 23% higher risk of local procedure-related complications, which is in accordance with our findings regarding the increased operative time required (80 min in group L vs. 135 min in group T). There was worse internal rotation in patients with a classic medialized Grammont prosthesis LDT in comparison to a lateralized RSTA alone. A review by Ortmaier et al. [19] even showed a complication rate of 26%, with *n* = 7 tendon-transfer-specific complications and 5 nerve palsies. Another possible disadvantage of tendon transfer is bony resorption. In 2022, Patel et al. published outcomes following RTSA with LDT [20]. This retrospective study included fifteen patients with preoperative ER lag. Twelve patients exhibited cortical humeral erosion at the latissimus dorsi transfer site. Bonnevialle et al. showed osteolysis in 77% of patients (2/13 minor, 1/13 moderate, and 7/13 major) [21]. And Kazum et al. showed osteolysis in 60% [17].

Despite the high incidence of radiologic changes after tendon transfer, no deleterious effects on short- to mid-term clinical outcomes were observed [17,20,21,22].

At 24 months postoperatively, the mean active internal rotation in our study did not show significant differences between groups (117° ± 26 in group L vs. 129° ± 26 in group T, *p* = 0.179). However, a significantly greater proportion of participants in the lateralized group (L) achieved a negative Apley scratch test—signifying enhanced functional internal rotation—compared to the medialized group with LDT (67% vs. 23%, *p* < 0.05). This indicates that, despite comparable angular range-of-motion values, lateralization without LDT may provide a clinically significant benefit in the recovery of functional internal rotation. No statistically or clinically significant changes were detected between the groups in terms of SPADI or Constant Scores, both being below the defined minimal clinically important difference (MCID) limits.

Although the mean active ER at 24 months was comparable between groups in our study, in line with the literature stating a mean gain of 29.4° external rotation after tendon transfer [23], group T exhibited a significantly greater improvement in external rotation from baseline, indicating a more pronounced rehabilitative effect. A considerably higher percentage of patients in group L achieved resolution of the ER lag sign at the final follow-up. These data suggest that although LDT may enhance the active external rotation range more effectively from a weak baseline, lateralized implants without tendon transfer may restore functional external rotation almost as well with a technically easier, less time-consuming procedure. Additionally, in group L, an intact teres minor had less impact on ER lag 24 months after surgery.

A study by Namdari et al. aimed at characterizing the functional range of motion of the shoulder and analyzed the movements required to proficiently perform 10 activities of daily living based on the functional evaluation elements of the ASES, PSS, and SST scoring systems. None of the chosen tasks evaluated external rotation with the arm positioned at the side. In 90° abduction, 60° of external rotation was necessary [24], while in other studies this value ranged from 21.8° to 91° [2,25].

Teres minor integrity also played a key role: although patients with a defective teres minor demonstrated greater ER gains postoperatively in our study—particularly in group T—the absence of this muscle severely limited the likelihood of ER lag resolution, especially in group T. In contrast, lateralized implants appeared to be less dependent on teres minor function, offering a more robust restoration of active ER control even in the setting of posterior cuff deficiency. But, as a limitation to this finding, we must acknowledge that we did not have clinical information on teres minor function, and therefore we cannot answer the question of whether a lateralized design without LDT would have been effective enough to reverse clinical signs of teres minor deficiency (high-grade ER lag sign >40° and hornblower sign) [26].

Radiographic analysis in our study indicated that increased prosthetic offset parameters were positively associated with enhanced postoperative external rotation and the resolution of the ER lag sign (*p* < 0.05). This highlights the biomechanical benefit of lateralization in the restoration of shoulder kinematics.

Several studies have demonstrated the effectiveness of lateralized RTSA in improving ER after its introduction by Frankle [27,28]. Lateralization of the COR has been shown to have positive effects on both internal rotation and ER in RTSA [8,9]. It allows a more beneficial lever arm, engaging the posterior aspect of the deltoid while maintaining residual functional ER of the rotator cuff [28,29]. The disadvantages of lateralized shoulder arthroplasties include increased shear forces [11]. Studies show the advantages of lateralization or the non-superiority of medialization [30]. But the role of the teres minor and the degree and side of lateralization remain controversial issues. Greiner et al. conducted an RCT comparing outcomes for patients who underwent RTSA using a lateralized design versus patients who received a standard design [29]. The lateralization group exhibited a significantly greater improvement in ER when patients with degenerative changes in the teres minor were excluded from the analysis. Conversely, research conducted by Berglund as well as Kwapisz et al. revealed no significant correlation between the atrophy of the teres minor and infraspinatus muscles and the restoration of ER in patients undergoing RTSA with a lateralized center of rotation [31,32].

Lateralization for reverse shoulder arthroplasty can be achieved in different ways. In our study, higher glenoidal offset proportionally translated to higher postoperative ER. The authors of this study would therefore suggest glenoidal lateralization, but more research with different types of lateralization needs to be carried out. The only prospective randomized study by Young et al. [33] compared CLEER patients who received RTSA with and without transfer. They found no significant differences in ADLER scores. But, as criticized by Boileau et al., there were some flaws in the patients’ selection, and there has been a critique of the use of the ADLER score and of preoperative values being missing, making a comparison difficult, as well as the fact that some patients were lost to follow-up. The heterogeneity of clinical data complicates the formulation of precise clinical indications for tendon transfers in RTSA. One other study directly compared a Grammont-style RTSA with a l’Episcopo tendon transfer with a lateralized RTSA: Merolla et al. conducted a study to evaluate the kinematic and electromyographic outcomes of two distinct designs for RTSA in ILER patients [34]. Thirteen Grammont-style RTSAs with l’Episcopo tendon transfers and twelve lateralized onlay RTSAs were compared. Indications for surgery were pseudoparalysis, cuff tear arthropathy, and a positive drop-arm sign. According to the study, RTSA featuring a lateralized humeral component achieved a restoration of active ER comparable to that observed in Grammont-style RTSA with l’Episcopo, suggesting that both approaches can be effective in addressing ER deficits in ILER patients.

Our study had some limitations. It was a retrospective study with a limited sample size (*n* = 34), constraining the statistical power and generalizability of our findings. While baseline demographic and clinical characteristics were similar across groups, the potential for selection bias remains a concern. The baseline data for active ER differed significantly between the two groups, and most procedures were conducted by two primary surgeons who treated patients in both groups, potentially introducing an element of unintentional preference or covert selection based on clinical nuances not entirely reflected in the dataset. Despite attempts to standardize surgical and rehabilitation protocols, minor variations in surgical decision-making may have impacted outcomes. In future studies a prospective, randomized controlled design will be utilized. Such a design would enhance control over confounding variables and improve the validity of comparisons between prosthesis types and the role of LDT in the management of rotator cuff tear arthropathy with severe ER deficiency. A significant limitation relates to the evaluation of the teres minor muscle. The study identified the status of the teres minor as a potential factor affecting postoperative external rotation and the presence of the ER lag sign; however, this assessment relied exclusively on preoperative imaging findings, lacking a formal clinical evaluation of teres minor function. Exclusive reliance on imaging may inadequately reflect the functional integrity or compensatory activity of muscle, which can fluctuate independently of structural appearance. It is possible that an intact teres minor tendon but of poor quality prevents patients from developing a full hornblower sign due to the “tenodesis” effect. Consequently, interpretations of the teres minor status’s role in elucidating outcome differences between groups must be approached with caution. Future research will integrate standardized clinical assessments of teres minor function with imaging techniques to enhance the overall evaluation. As mentioned, we want to emphasize again the fact that we did not address all the CLEER criteria as we did not have information on hornblower or drop signs and did not exclude a priori Goutallier 2 teres minor muscle status; however, there was a similar proportion of teres minor third- and fourth-grade fatty infiltration in both groups (42% vs. 46%), and the overall Goutallier status did not differ between the groups. The assessment of the ER lag sign was not standardized across all evaluators, and the degree of lag was not graded using a validated scoring system. This may have introduced variability in how the presence and severity of the sign were recorded, potentially affecting group comparisons.

## 5. Conclusions

To conclude, our main finding was that the utilization of a lateralized prosthetic design is nearly as effective and technically easier in treating patients with CTA and external rotation deficit compared to a medialized prosthesis with LDT, while latissimus dorsi transfer certainly resulted in a significantly greater change from baseline. Furthermore, a lateralized prosthesis shows significantly less loss of internal rotation compared to a medialized prosthesis with LDT.

## Figures and Tables

**Table 1:** Baseline demographics, preoperative clinical characteristics, and radiographic prosthetic offset parameters.

Baseline Parameters	Group L (*n*)	Mean (SD)	Median (Range)	Group T (*n*)	Mean (SD)	Median (Range)	SMD
**Age at surgery**	21	78 (7)	79 (62 to 87)	13	69 (6)	69 (57 to 77)	1.425
**Age (3 classes) (*n*, %)**							1.65
< 70	3 (14)			9 (69)			
70–80	9 (43)			4 (31)			
>80	9 (43)						
**Sex (*n*, %)**							0.107
Female	14 (67)			8 (62)			
Male	7 (33)			5 (38)			
**Diagnosis (*n*, %)**							0.459
CTA	21 (100)			13 (100)			
**Classification of arthrosis acc. to Samilson**							0.76
I	4 (36)			8 (62)			
II	5 (45)			5 (38)			
III	2 (18)						
**Classification of arthropathy acc. to Hamada**							0.768
Stage 1	5 (45)			3 (23)			
Stage 2	2 (18)			1 (8)			
Stage 3	1 (9)			2 (15)			
Stage 4A				1 (8)			
Stage 4B	3 (27)			6 (46)			
**Fatty infiltration of teres minor Goutallier 3/4 in %**	42			46			
**Flexion (active) (°)**	21	73 (43)	70 (0 to 170)	13	87 (52)	80 (10 to 170)	0.294
**Abduction (active) (°)**	21	68 (35)	70 (0 to 130)	13	86 (45)	70 (20 to 170)	0.433
**External rotation (active) (°)**	21	0 (0)	0 (0 to 0)	12	−13 (12)	−15 (−30 to 0)	1.58
**Internal rotation (active) (*n*, %)**							0.8
Lat. thigh	1 (5)						
Gluteal region	3 (14)			1 (8)			
Lumbosacral region	2 (10)			1 (8)			
L3	10 (48)			6 (46)			
Th12	3 (14)			5 (38)			
Th7	2 (10)						
**Flexion (passive) (°)**	21	101 (42)	90 (15 to 170)	13	115 (45)	125 (40 to 180)	0.306
**Abduction (passive) (°)**	21	92 (33)	80 (50 to 170)	13	113 (42)	115 (50 to 180)	0.553
**External rotation (passive) (°)**	21	20 (14)	20 (0 to 55)	13	26 (14)	20 (10 to 60)	0.422
**Abduction force (kg)**	21	0.5 (1.2)	0.0 (0.0 to 4.0)	13	0.8 (1.3)	0.0 (0.0 to 4.0)	0.289
**Constant–Murley Score (0 = min 100 = max)**	19	30 (16)	26 (3 to 64)	13	37 (14)	34 (21 to 72)	0.45
**SPADI (0 = worst, 100 = best)**	21	35 (23)	31 (6 to 82)	12	42 (22)	41 (13 to 86)	0.292
**Medialization of center of rotation (mm)**	12	20.4 (4.4)	20.4 (14.6 to 27.9)	12	23.7 (2.8)	24.1 (19.4 to 29.1)	1.97
**Humeral offset (mm)**	20	22.0 (3.9)	21.5 (16.0 to 30.6)	12	17.4 (3.1)	17.6 (12.7 to 21.7)	0.733
**Glenoidal offset (mm)**	20	5.4 (3.2)	5.1 (1.0 to 16.9)	11	0.3 (1.0)	0.3 (−1.9 to 2.1)	0.988
**Glenosphere offset (mm)**	19	18.4 (1.7)	18.0 (14.0 to 21.0)	12	15.3 (1.5)	14.0 (14.0 to 17.0)	0.105

**Table 2:** Postoperative clinical outcomes at 24-month follow-up.

Parameters	Group L (*n*)	Mean (SD)	Median (Range)	Group T (*n*)	Mean (SD)	Median (Range)	*p*-Value
**Flexion (active)**	10	133 (22)	125 (110 to 170)	13	134 (22)	140 (80 to 170)	0.57
**Abduction (active)**	10	117 (26)	115 (90 to 160)	13	129 (26)	130 (70 to 160)	0.23
**External rotation (active)**	10	19 (10)	20 (0 to 30)	13	22 (7)	20 (10 to 30)	0.35
**External rotation (active) change from baseline**	10	18.5(9.5)	20(0 to 30)	13	34.6 (13.1)	35 (10 to 55)	<0.05
**Internal rotation (active)**							0.16
**Lat. thigh**	0 (0)			0 (0)			
**Gluteal region**	2 (20)			7 (54)			
**Lumbosacral region**	2 (20)			3 (23)			
**L3**	2 (20)			3 (23)			
**Th12**	2 (20)						
**Th7**	2 (20)						
**Constant–Murley Score (0 = min 100 = max)**	8	63 (9)	65 (45 to 73)	11	63 (16)	63 (29 to 83)	0.93
**Abduction force (kg)**	10	3.9 (1.7)	3.8 (1.0 to 7.0)	12	5.5 (2.9)	5.0 (0.0 to 10.0)	0.11
**SPADI (0 = worst, 100 = best)**	16	74 (22)	80 (23 to 99)	10	73 (20)	78 (25 to 91)	0.9
**ER lag sign (*n*, %)**							<0.05
**Negative**	7 (70)			3 (23)			
**Positive**	3 (30)			10 (77)			
**Apley’s test internal rotation up to L3 or more (*n*, %)**							<0.05
**No**	3 (33)			10 (77)			
**Yes**	6 (67)			3 (23)			

**Table 3:** Radiographic prosthetic offset parameters per ER lag status.

Parameter	Negative ER Lag (*n*)	Mean (SD)	Median (Range)	Positive ER Lag (*n*)	Mean (SD)	Median (Range)	Effect (95% CI)	*p*-Value
**Humeral Offset**	18	21.3 (4.0)	19.5 (16.9 to 28.4)	16	17.7 (3.2)	18.5 (12.7 to 21.7)	−3.6 (−6.6 to −0.6)	<0.05
**Glenoidal Offset**	18	5.6 (5.0)	5.3 (0.0 to 16.9)	16	1.1 (2.1)	0.6 (−1.9 to 5.3)	−4.5 (−7.6 to −1.4)	<0.05
**Glenosphere Offset**	18	17.2 (1.6)	18.0 (14.0 to 19.5)	16	15.5 (2.0)	14.0 (14.0 to 19.5)	−1.7 (−3.3 to −0.1)	0.055

## Data Availability

The original contributions presented in this study are included in the article. Further inquiries can be directed to the corresponding author(s).
